# Association between depression and the prevalence and prognosis of prediabetes: Data from National Health and Nutrition Examination Survey (NHANES) 2013–2018

**DOI:** 10.1371/journal.pone.0304303

**Published:** 2025-01-13

**Authors:** Jin Zhou, Xiaojiao Yang

**Affiliations:** Department of Pharmacy, NHC Key Laboratory of Hormones and Development, Tianjin Key Laboratory of Metabolic Diseases, Chu Hsien-I Memorial Hospital & Tianjin Institute of Endocrinology, Tianjin Medical University, Tianjin, China; University of Montenegro-Faculty of Medicine, MONTENEGRO

## Abstract

**Background:**

Diagnosis and intervention of prediabetes is an emerging approach to preventing the progression and complications of diabetes. Inflammatory factors and dysregulation of the hypothalamic-pituitary-adrenal (HPA) axis have been suggested as potential mechanisms underlying the pathogenesis of both diabetes and depression. However, the relationship between depression levels and the prevalence of prediabetes and its prognosis remains elusive. This study aimed to explore the relationship between depression and the prevalence of prediabetes and to further explore the all-cause mortality of different levels of depression in patients with prediabetes.

**Methods:**

Our study used a data set from the National Health and Nutrition Examination Survey (NHANES). Participants were initially divided into two groups (depression vs. non-depression) and further stratified by different depression severity levels. We used a weighted multiple logistic regression model to analyze the association between depression and prediabetes prevalence and a Cox regression model to assess all-cause mortality in prediabetic patients.

**Results:**

A total of 4384 participants were included, divided into depression group (n = 1379) and non-depression group (n = 3005). Results showed that people with depression were at higher risk of developing prediabetes. After adjusting for covariates, moderate to severe depression was positively associated with prediabetes (moderate to severe depression vs no depression: OR = 1.834, 95%CI: 0.713–4.721; severe depression vs no depression: OR = 1.004, 95% CI 0.429–2.351). In addition, we explored the relationship between all-cause mortality and depressive status in patients diagnosed with prediabetes (n = 2240) and found that moderate to severe depression (HR = 2.109, 95%CI 0.952–4.670) was associated with higher mortality in patients with prediabetes.

**Conclusions:**

Overall, the findings consistently suggest that depression is positively associated with both the prevalence and mortality risk among individuals with prediabetes. This suggests that depression may be a new and valuable indicator of prediabetes risk. Early treatment of depression improves outcomes in prediabetes.

## Introduction

Prediabetes is a stage of moderate hyperglycemia with specific parameters including impaired fasting glucose, impaired glucose tolerance (IGT) [1], and specific ranges of hemoglobin A1c (HbA1c) as defined by the American Diabetes Association (ADA) [2]. Prediabetes not only serves as a precursor to type 2 diabetes but is also associated with a range of serious health issues, including cardiovascular diseases, neurological complications, and mental health problems. Studies have shown that individuals with prediabetes and type 2 diabetes frequently experience cerebral microvascular complications, such as stroke, cognitive dysfunction, and depression [3]. A recent study suggests that early management of prediabetes can reduce the risk of progression to diabetes [4]. Despite this, prediabetes remains under-recognized and under-researched compared to diabetes. Therefore, it is necessary to identify more early intervention measures to prevent the progression of prediabetes to diabetes and the occurrence of its complications.

Patients with diabetes live longer, the risks and burdens of emerging complications of diabetes begin to come into view [5]. Studies have shown that patients with diabetes have a 25% increased risk of depression [6]. A large-scale prospective study investigating whether diabetic complications increase the risk of depression and/or anxiety among adults with type 2 diabetes included 265,799 individuals diagnosed with type 2 diabetes between 1997 and 2017, with no recent history of depression or anxiety. The study found that the incidence rate of depression and/or anxiety was 3,368 per 100,000 person-years among individuals with diabetic complications, compared to 1,929 per 100,000 person-years among those without complications. The presence of any diabetic complication was associated with an increased risk of depression and/or anxiety (HR 1.77, 95% CI 1.73–1.80). All types of diabetic complications increased the risk of depression and/or anxiety [7]. However, there is no clear evidence indicating whether depression increases the risk of diabetes. Current perspectives suggest that inflammatory factors and HPA axis dysfunction may be the underlying pathogenesis of diabetes and depression [8, 9]. Nevertheless, it remains to be determined whether patients with depression are more likely to develop prediabetes and face an increased risk of mortality.

Currently, there is no effective way to avoid the development of prediabetes. Therefore, we tried to find early markers of prediabetes and intervene accordingly. Based on the relationship between mental health conditions and glycemic control, we hypothesized that depression would be associated with a higher prevalence of prediabetes and a worse prognosis. Therefore, the purpose of this study was to determine whether depression is a risk factor for the incidence of prediabetes and adverse outcomes using data from the National Health and Nutrition Examination Survey (NHANES).

## Materials and methods

### Study population

NHANES is a continuous survey designed to assess the health and nutritional status of adults and children in the United States using a complex, multistage probability sampling method. The data for this study were obtained from the 2013–2018 NHANES cycles. All procedures were conducted following the ethical standards set by the National Center for Health Statistics (NCHS) Ethical Review Board and in accordance with the 1975 Declaration of Helsinki, revised in 2013.

### Identification of prediabetes and depressive states

The diagnosis of prediabetes is based on one of the following four principles [10]: (1) Self-reported prediabetes: Participants answered “Have you ever been told about prediabetes/IGT/IFG/borderline diabetes/blood sugar higher than normal, but not high enough? Known as the Diabetes Questionnaire. (2) HbA1c: 5.7%–7.0%. (3) Fasting blood glucose: 5.6–7.0 mmol/L. (4) Oral glucose tolerance test: 7.8–11.0 mmol/L.

The PHQ-9 health questionnaire (Patient Health Questionnaire-9, PHQ-9) is based on 9 items of the diagnostic criteria of DSM-IV (Diagnostic and Statistical Manual of Mental Disorders developed by the American Psychiatric Association) [11]. A simple and effective self-rating scale for depressive disorder. It has good reliability and validity in assisting the diagnosis of depression and assessing the severity of symptoms. Participants answered this questionnaire based on the situation in the past two weeks. The answers to each question From left to right are "rarely", "a few days", "more than half", and "almost every day". The scores of the corresponding options are: 0, 1, 2, 3. Add each question to get the final score.

### Inclusion and exclusion process

**Fig 1:**
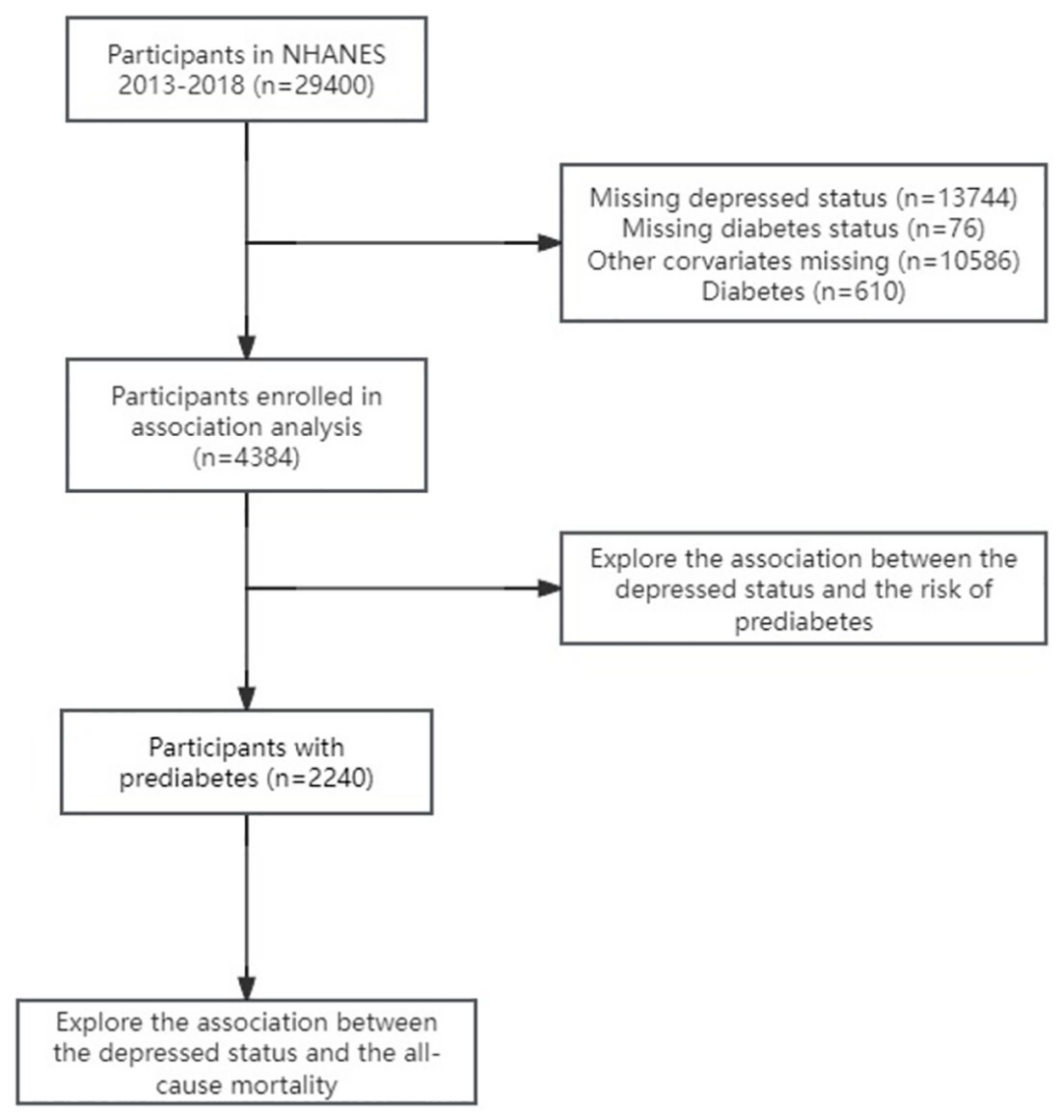
Study workflow.

We used data from the NHANES 2013–2018 cycle. First, we included 4384 patients with clear data on depression status, prediabetes, and other covariates to explore the relationship between depression status and risk of prediabetes. In addition, we also recruited 2240 prediabetic patients from a sample of 4384 to further explore the relationship between depressive status and all-cause mortality (Fig 1).

### Outcomes

The result of the correlation analysis was a diagnosis of prediabetes. The outcome of the survival analysis was mortality among patients diagnosed with depression in prediabetes. All-cause mortality was determined through linkage to National Death Index records for 2019. ICD-10 is used to determine cause of death.

### Assessment of covariates

A standardized questionnaire obtained information on sociodemographic characteristics, smoking status, and alcohol consumption. Individuals who had smoked >100 cigarettes in their lifetime were considered current smokers, whereas those who had smoked >100 cigarettes and had quit smoking were considered former smokers. A never-drinker does not drink alcohol every year, a light drinker drinks 1–11 times a year, a moderate drinker drinks 1–3 times a month, and a moderate drinker drinks 1–4 times a week. Heavy drinkers are those who drink alcohol almost every day. Body mass index (BMI) is calculated from weight/height^2^ (kg/m^2^). The diagnosis of diabetes is as follows: (1) The doctor diagnoses you as having diabetes; (2) hemoglobin A1c>7%; (3) Fasting blood glucose ≥7.0 mmol/L; (4) Random blood glucose ≥11.1 mmol/L, (5) Oral administration within 2 hours Glucose tolerance test (OGTT) blood glucose ≥11.1 mmol/L; (6) Use of diabetes drugs or insulin. Hypertension, hyperlipidemia, and coronary heart disease (CHD) were identified as relevant diseases with “yes” responses to the questionnaire. In addition, the researchers conducted relevant laboratory data analysis, including assessment of baseline glucose, insulin, hemoglobin A1c, total cholesterol, triglycerides, high-density lipoprotein, and low-density lipoprotein levels.

### Statistical analysis

All analyses incorporate sample weights, strata, and primary sampling units to produce accurate national estimates. Sample characteristics are reported as means (SEs) for normally distributed continuous variables, medians for non-normally distributed continuous variables, and percentages for categorical variables. Using the no-depression group as the reference, a weighted multivariable logistic regression model was used to estimate the OR and 95% confidence interval associated with depressive status and prediabetes. Weighted Cox proportional hazards regression was used to estimate hazard ratios (HRs) and 95% CIs for all-cause mortality associated with depressive status. Person-time was calculated as the time interval between the NHANES interview date and the date of death or end of follow-up (December 31, 2019), whichever occurred more frequently. We constructed three statistical models. Model 1: No adjustment for covariates. Model 2: For age (continuous), sex (male or female), race/ethnicity (Mexican American, other Hispanic, non-Hispanic white, non-Hispanic black, or other race), and education level (less than high school, High school, high school and above) have been adjusted. Model 3: Age (continuous), sex (male or female), race/ethnicity (Mexican American, other Hispanic, non-Hispanic white, non-Hispanic black, or other race), education level (less than high school, high school, high school or above), smoking status (former or current), alcohol consumption (never, light, moderate, moderate to severe, severe), BMI (continuous), hypertension (yes or no), hyperlipidemia (yes or no)) and coronary heart disease (yes or no) were adjusted. Individuals with missing covariate data in Model 2 were not included in the analyses of missingness in Model 2 and Model 3 (listwise deletion). Additionally, stratified analyses were performed to examine whether the detected associations differed by age, sex, and body mass index. All analyses were performed using R software (4.1.0). A two-sided *p*<0.05 was considered statistically significant.

## Results and discussion

### Participant characteristics

**Fig 2:**
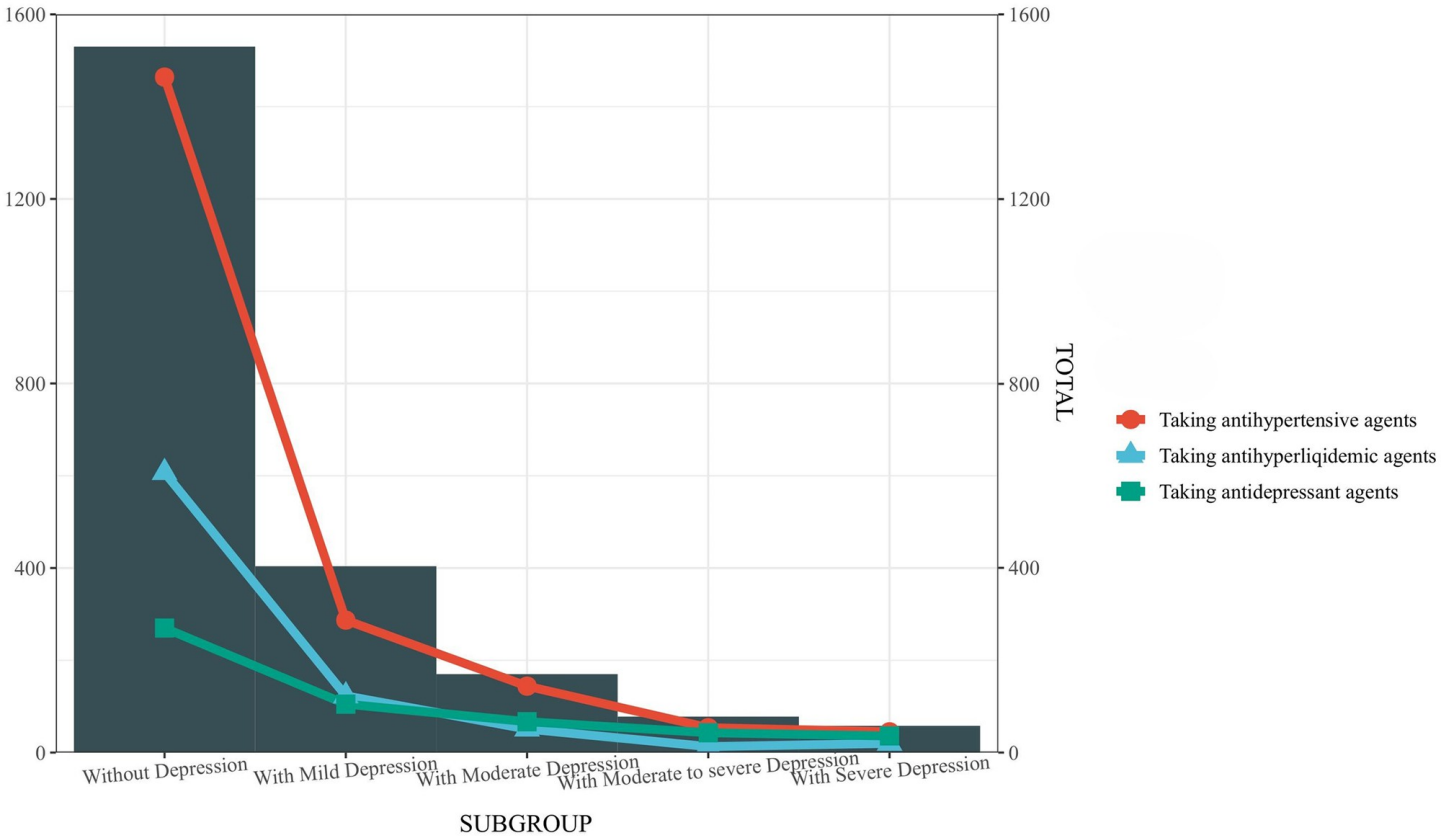
The use of antihypertensive agents, antihyperlipidemic agents and antidepressant agents in patients with different depressive status.

In this study, 4384 participants participated in our analyses. The patients were 1379 patients with depression and 3005 patients without depression. Compared with participants without depression, participants with depression were more likely to be female, have lower education, have hypertension, hyperlipidemia, moderate to heavy alcohol use, current smoking, higher BMI, and have diabetes Early stage (Table 1). Among participants without depression, the use of antihypertensive drugs, antihyperlipidemic drugs, and antidepressants was 95.68%, 39.67%, and 17.64%, respectively, and we stratified participants with depression, it was found that the use rate of antihypertensive drugs was the highest in moderate depression (84.70%), and the use rate of antihyperlipidemic drugs and antidepressants was the highest in severe depression (34.48%, 62.06%) (Fig 2).

**Table 1:** Baseline characteristics of included participants in NHANES 2013–2018.

Variables	Total-4384	Without Depression -3005	With Depression -1379	*p*-value
Age, years	51.13±16.71	51.60±15.68	50.11±16.11	0.005
Gender, %				<0.001
Female	1774(40.46%)	1058(35.21%)	716(51.92%)	
Male	2610(59.54%)	1947(64.79%)	663(48.08%)	
Race, %				<0.001
Non-Hispanic White	2069(47.19%)	1415(47.09%)	654(47.42%)	
Non-Hispanic Black	936(21.35%)	646(21.50%)	290(21.03%)	
Mexican American	491(11.20%)	344(11.45%)	147(10.66%)	
Other Race	509(11.61%)	349(11.61%)	160(11.61%)	
Other Hispanic	379(8.65%)	251(8.35%)	128(9.28%)	
Education level, %				<0.001
Less than high school	894(20.39%)	560(18.64%)	334(24.22%)	
High school	1176(26.83%)	790(26.29%)	386(27.99%)	
More than high school	2314(52.78%)	1655(55.07%)	659(47.79%)	
Prediabetes, %				<0.001
No	2144(48.91%)	1475(49.08%)	669(48.51%)	
Yes	2240(51.09%)	1530(50.92%)	710(51.49%)	
Hypertension, %				<0.001
No	2759(62.93%)	1958(65.16%)	801(58.09%)	
Yes	1625(37.07%)	1047(34.84%)	578(41.91%)	
CHD, %				0.142
No	4185(95.46%)	2878(95.77%)	1307(94.78%)	
Yes	199(4.54%)	127(4.23%)	72(5.22%)	
Alcohol use, %				<0.001
Never	392(8.94%)	249(8.29%)	143(10.37%)	
Mild	411(9.38%)	261(8.69%)	150(10.88%)	
Moderate	327(7.46%)	236(7.85%)	91(6.60%)	
Moderate to severe	1130(25.77%)	751(24.99%)	379(27.48%)	
Severe	2124(48.45%)	1508(50.18%)	616(44.67%)	
Smoke status, %				<0.001
Former	2259(51.53%)	1679(55.87%)	580(42.06%)	
Now	2125(48.47%)	1326(44.13%)	799(57.94%)	
BMI	29.17±7.04	28.72±6.50	30.16±8.02	<0.001
Triglyceride, mmol/L	1.29±0.95	1.27±0.97	1.33±0.90	0.163
Cholesterol, mmol/L	4.94±1.08	4.93±1.07	4.94±1.11	0.976
HDL, mmol/L	1.38±0.44	1.39±0.44	1.36±0.45	0.018
LDL, mmol/L	2.92±0.91	2.91±0.91	2.92±0.93	0.732
Fasting blood glucose, mmol/L	5.59±0.57	5.60±0.56	5.57±0.59	0.440
Insulin, pmol/L	69.29±89.25	66.15±94.36	76.50±75.70	0.005
HbA1c	5.51±0.44	5.51±0.43	5.52±0.45	0.975
Postprandial blood glucose, mmol/L	5.96±1.79	5.90±1.78	6.11±1.85	0.773

### Logistic regression between depression and prediabetes

We used a weighted multivariable logistic regression model to explore the relationship between depression and prediabetes (Table 2). Compared with non-depression, depression was positively associated with prediabetes in all models (OR = 1.023, 95% CI 0.901–1.162; 1.117, 95% CI 0.976–1.279; 1.016, 95% CI 0.881–1.172). All models have *p*-values less than 0.001. According to the PHQ-9 Health Questionnaire, we further divided depressive states into 5 types. In model 1, mild, moderate to severe, severe depressive states were significantly associated with prediabetes (OR = 1.065, 95% CI 0.735–1.544; 1.648, 95% CI 0.719–3.777; 1.348, 95% CI 0.617–2.945). The *p*-values for all these depression states were less than 0.001. After adjusting for covariates, in model 2 (OR = 1.065, 95% CI 0.735–1.544; 2.076, 95% CI 0.831–5.185) and model 3 (1.834, 95% CI 0.713–4.721; 1.004, 95%CI 0.429–2.351), moderate to severe and severe depression were positively associated with prediabetes. Furthermore, associations remained between depression and prediabetes in most subgroups.

**Table 2:** Association between prediabetes and depression among participants in NHANES 2013–2018.

Depress status	Model 1 OR(95% CI) *p* value	Model 2 OR(95% CI) *p* value	Model 3 OR(95% CI) *p* value
Depression			
No	1	1	1
Yes	1.023 (0.901,1.162)	1.117 (0.976,1.279)	1.016 (0.881,1.172)
	<0.001	<0.001	<0.001
Depression by PHQ-9			
No	1	1	1
Mild	1.065 (0.735,1.544)	1.050 (0.711,1.551)	1.177 (0.785,1.764)
	<0.001	0.268	0.431
Moderate	0.922 (0.560,1.518)	0.850 (0.503,1.439)	1.029 (0.594,1.782)
	<0.001	<0.001	<0.001
Moderate to severe	1.648 (0.719,3.777)	1.065 (0.735,1.544)	1.834 (0.713,4.721)
	<0.001	<0.001	0.007
Severe	1.348 (0.617,2.945)	2.076 (0.831,5.185)	1.004 (0.429,2.351)
	<0.001	<0.001	<0.001

Model 1: no covariates were adjusted. Model 2: age (continuous), gender (male or female) and race/ethnicity (Mexican American, other Hispanic, non-Hispanic white, non-Hispanic black, or other Race), education level (less than high school, high school, more than high school) were adjusted. Model 3: age (continuous), gender (male or female) and race/ethnicity (Mexican American, other Hispanic, non-Hispanic white, non-Hispanic black, or other Race), education level (less than high school, high school, more than high school), smoking status (former, or current), alcohol use (never, mild, moderate, moderate to severe, severe), BMI (continuous), hypertension (no or yes), hyperlipidemia (yes or no), CHD (yes or no) were adjusted.

The PHQ-9 Health Questionnaire (Patient Health Questionnaire-9, PHQ-9) is based on nine items of the diagnostic criteria of DSM-IV (Diagnostic and Statistical Manual of Psychiatric Disorders developed by the American Psychiatric Society)

### Cox regression analysis between depression and mortality in patients with prediabetes

**Fig 3:**
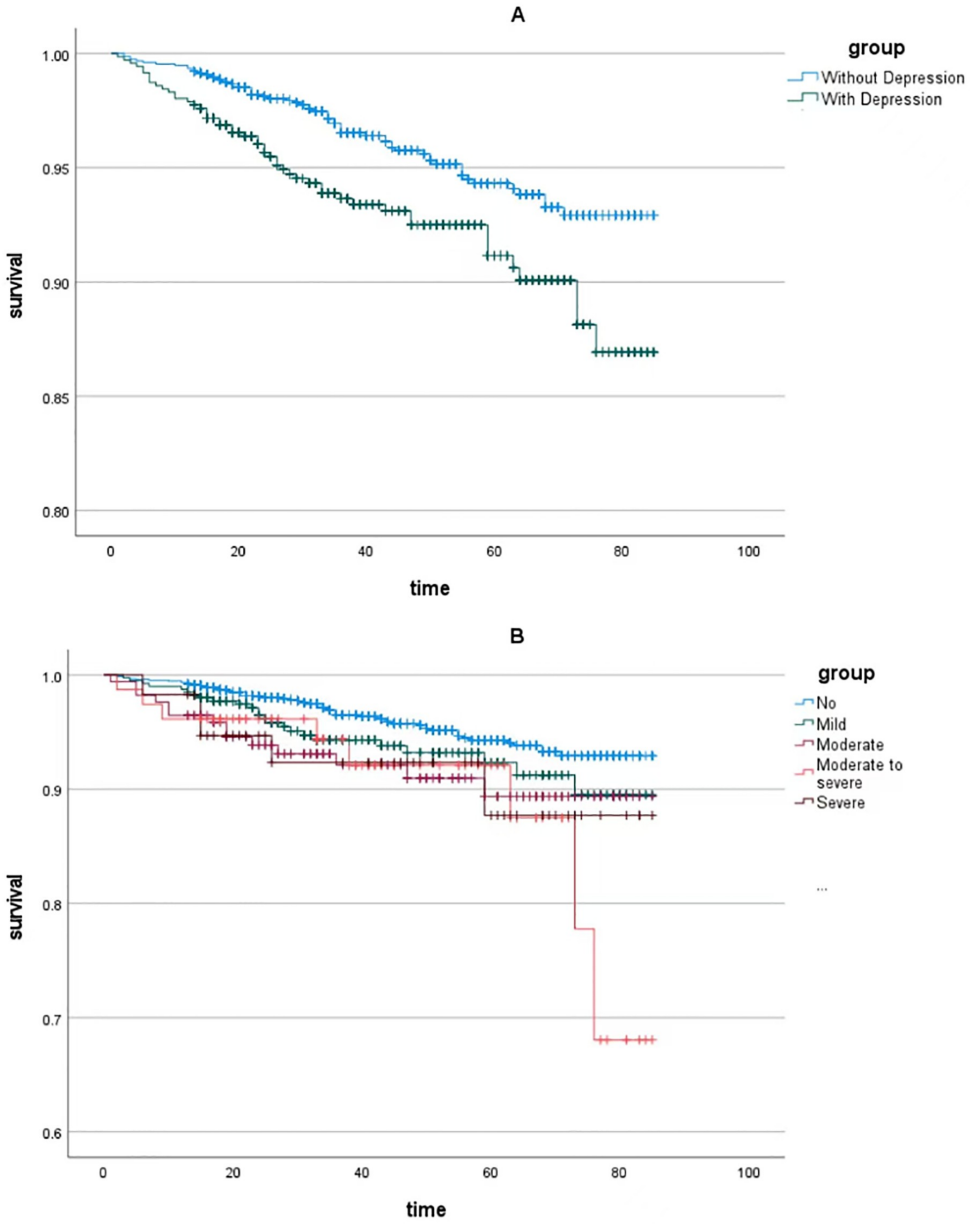
Kaplan‒Meier survival curve of prediabetic patients with/without depression A. Kaplan‒Meier survival curve of the prediabetes patients with/without depression; B. Kaplan‒Meier survival curve of the prediabetes patients with different statuses of depression.

A total of 2240 patients with prediabetes were included, and the weighted Cox proportional hazards regression method was used to further explore the correlation between depression status and mortality in patients with prediabetes. The average follow-up time was 44.47±43.84 months. Compared with the non-depressed group, patients with depressed status were significantly associated with mortality in prediabetes in all models (HR = 1.570, 95% Cl 1.180–2.088, *p* = 0.002; 1.816, 95% Cl 1.359–2.427, *p* <0.001; 1.708, 95% Cl 1.273–2.290, *p*<0.001). After stratifying by depression status, moderate and moderate-severe depression were positively associated with prediabetes mortality (HR = 1.946, 95% Cl 1.069–3.542, *p* = 0.029; 2.682, 95% Cl 1.283–5.605, *p* = 0.009). After further adjusting for age, gender, race, education level, drinking status, smoking status, BMI, hypertension, hyperlipidemia, and coronary heart disease, moderate to severe depression was associated with model 2 (HR = 2.459, 95% CI 1.118–5.407, *p* = 0.025) and model 3 (HR = 2.109, 95%CI 0.952–4.670, *p* = 0.032) were related to prediabetes mortality (Table 3), and the Kaplan-Meier survival curve also obtained similar results (Fig 3).

**Table 3:** All-cause mortality of patients with prediabetes during different depress status in NHANES 2013–2018.

Depress status	Model 1	Model 2	Model 3
	HR(95% CI) *p* value	HR(95% CI) *p* value	HR(95% CI) *p* value
Depression			
No	1	1	1
Yes	1.570 (1.180,2.088)	1.816 (1.359,2.427)	1.708 (1.273,2.290)
	0.002	<0.001	<0.001
Depression by PHQ-9			
No	1	1	1
Mild	1.570 (0.951,2.482)	1.755 (1.082,2.847)	1.796 (1.1032.924)
	0.079	0.023	0.018
Moderate	1.946 (1.069,3.542)	1.982 (1.061,3.702)	1.745 (0.926,3.290)
	0.029	0.066	0.085
Moderate to severe	2.682 (1.283,5.605)	2.459 (1.118,5.407)	2.109 (0.952,4.670)
	0.009	0.025	0.032
Severe	1.707 (0.621,4.695)	2.249 (0.804,6.289)	1.850 (0.656,5.217)
	0.300	0.122	0.245

Model 1: no covariates were adjusted. Model 2: age (continuous), gender (male or female) and race/ethnicity (Mexican American, other Hispanic, non-Hispanic white, non-Hispanic black, or other Race), education level (less than high school, high school, more than high school) were adjusted. Model 3: age (continuous), gender (male or female) and race/ethnicity (Mexican American, other Hispanic, non-Hispanic white, non-Hispanic black, or other Race), education level (less than high school, high school, more than high school), smoking status (former, or current), alcohol use (never, mild, moderate, moderate to severe, severe), BMI (continuous), hypertension (no or yes), hyperlipidemia (yes or no), CHD (yes or no) were adjusted.

The PHQ-9 Health Questionnaire (Patient Health Questionnaire-9, PHQ-9) is based on nine items of the diagnostic criteria of DSM-IV (Diagnostic and Statistical Manual of Psychiatric Disorders developed by the American Psychiatric Society)

Depression is a common mental illness. Its main characteristics include low mood, reduced interest, pessimism, slow thinking, lack of initiative, self-blame, poor diet, and sleep, worry about suffering from various diseases, and feeling that the whole body is tired. Feeling unwell, and in severe cases, suicidal thoughts and behaviors may occur. Maina et al. [12] reported that there is a causal relationship and a positive correlation between depression and type 2 diabetes. Prediabetes is a state in which blood sugar levels have increased but have not yet reached the diagnostic criteria for diabetes. During this stage, an individual’s risk of developing diabetes increases. To explore this relationship further, our study aimed to examine the relationship between depression and prediabetes. The results of this study indicate that moderate to severe depression is associated with the development of prediabetes. Nevertheless, the results fail to demonstrate a dose-response effect, as severe depression is not associated with higher odds of prediabetes compared to moderate to severe depression. In this study we calculated HOMA-IR (homeostasis model assessment of insulin resistance index) levels in addition to the assessment of fasting blood glucose and fasting insulin levels in each group of people, it was found that the insulin resistance index of the non-depression group was lower than that of the Depression Group.

HOMA-IR was first University of Oxford in 1985 by Turner’s team to assess an individual’s level of insulin resistance. The formula was HOMA-IR = fasting plasma glucose (FPG, mmol/L) × fasting insulin (FINS, μu/mL)/22.5. The HOMA-IR index of normal individuals is about 1, and with the increase of insulin resistance, the HOMA-IR index will be higher than 1. In the treatment of diabetes and metabolic syndrome, HOMA-IR can be used to assess the efficacy of drugs, such as the improvement of insulin resistance by certain glucose-lowering drugs and insulin sensitizers. Although HOMA-IR has extensive application value as a substitute marker of insulin resistance, it still has some limitations. For example, HOMA-IR calculations rely on fasting blood glucose and fasting insulin levels, which may be influenced by a variety of factors, such as stress status, drug interference, and so on. Therefore, in the application of HOMA-IR, the need to integrate the specific situation of patients with a comprehensive analysis [13].

Other studies [14–16] reported that the incidence of anxiety and depression in patients with prediabetes is higher than that in the normal population. As early as 2016, Liu et al. [17] established a one-year China Health and Retirement Longitudinal Study (CHARLS), which included Chinese elderly with known or unknown diabetes. Participants with newly diagnosed diabetes or prediabetes were considered to have unknown diabetes. Findings showed that knowledge of having diabetes, treatment, and other chronic conditions was associated with higher rates of depression in patients with known diabetes compared with those with unknown diabetes. The prevention of depression in patients with diabetes should receive more attention among middle-aged, female, and less educated people. Some of the findings are similar to ours in that we used the PHQ-9 criterion to stratify depressive status, which significantly contributes to the development of depression. Therefore, large prospective studies are still needed to study the impact of depression on the development and prognosis of prediabetes. Tricyclic antidepressants may affect the function of Islet β-cells, lead to abnormal insulin secretion, and promote the development of prediabetes. Selective serotonin reuptake inhibitor (SSRIs): although these drugs do not usually increase the risk of diabetes significantly, long-term or high-dose use may still have an effect on glucose metabolism. Studies have shown that long-term use of large doses of antidepressants in patients with a higher risk of type 2 diabetes than the non-antidepressant population. In addition, discontinuation or reduction of antidepressant doses can improve glucose tolerance in diabetic patients. In addition to the effects of the drug itself, the patient’s age, weight, family history, lifestyle and other factors may also increase the risk of prediabetes. Therefore, multiple factors need to be taken into account when evaluating the relationship between antidepressants and prediabetes. Regular monitoring of blood glucose levels and related metabolic indicators is recommended for patients who are taking antidepressants, especially those with a family history of diabetes or other risk factors. Doctors should choose appropriate antidepressant drugs and doses according to the specific conditions of patients, and pay close attention to patients’ weight changes and glucose metabolism. When necessary, the type or dose of the drug can be adjusted to optimize efficacy and safety. Encourage patients to maintain a healthy lifestyle, including a balanced diet, moderate exercise and good mental health. These measures help to reduce the risk of prediabetes and improve the overall health of patients. Regular monitoring of blood glucose levels and related metabolic indicators is recommended for patients who are taking antidepressants, especially those with a family history of diabetes or other risk factors. Doctors should choose appropriate antidepressant drugs and doses according to the specific conditions of patients, and pay close attention to patients’ weight changes and glucose metabolism. When necessary, the type or dose of the drug can be adjusted to optimize efficacy and safety. Encourage patients to maintain a healthy lifestyle, including a balanced diet, moderate exercise and good mental health. These measures help to reduce the risk of pre-diabetes and improve the overall health of patients [18].

Surprisingly, several other studies that did not look at the association between prediabetes and depression also found a potential relationship. Type 2 diabetes is associated with cerebral small vessel complications such as stroke, cognitive impairment, and depression [19–22]. After stratifying by depression status, moderate and moderate-to-severe depression were positively associated with prediabetes mortality (HR = 1.946, 95% CI 1.069–3.542, *p* = 0.029; 2.682, 95% CI 1.283–5.605, *p* = 0.009). After further adjusting for age, gender, race, education level, drinking status, smoking status, BMI, triglycerides, HDL, insulin, hypertension, hyperlipidemia, and coronary heart disease, moderate-to-severe depression remained associated with prediabetes mortality. These conditions underscore the interplay between metabolic and mental health disorders, highlighting the necessity for comprehensive management strategies addressing both aspects. Evidence has shown abnormalities in H-reflex and H-reflex dependent inhibition in patients with diabetes, prediabetes, and obesity [23]. These neuromuscular abnormalities provide further insight into the physiological impact of metabolic disorders and their potential contribution to mental health issues. Studies such as the CARYATID study have shown that elderly prediabetic patients with hypertension and chronic kidney disease are at an increased risk of frailty and cognitive impairment [24]. This underscores the importance of early detection and intervention in this vulnerable population to prevent further organ damage and cognitive decline. The association between depression and cardiovascular risk is well-documented. Depressive symptoms, inflammatory markers, and cardiovascular risk factors, such as PCSK9 levels and insulin resistance, are interconnected [25, 26]. This relationship emphasizes the need for integrated treatment approaches that address both mental and physical health in prediabetic patients. The CENTENNIAL study highlights the impact of insulin resistance on cognitive impairment in hypertensive elderly prediabetic patients [27]. This finding points to the importance of managing insulin resistance to prevent cognitive decline and improve overall health outcomes in this population. Childhood abuse has been identified as an independent risk factor for dysregulated glucose metabolism in prediabetes [28]. This highlights the long-term impact of early life stress on metabolic health and the need for targeted interventions to address these effects. Increased frailty risk has been observed in hypertensive elderly prediabetic patients. Metformin has shown beneficial effects in reducing this risk, providing a potential preventive strategy for managing prediabetes in this high-risk group [29]. Routine HbA1c testing in psychiatric inpatients has been used to assess glycemic status, emphasizing the importance of regular monitoring to detect prediabetes early in patients with mental health disorders [30]. Understanding patients’ cognition about diabetes and prediabetes is crucial for developing effective educational and intervention strategies. Depression can affect patients’ awareness and beliefs about their health conditions, influencing their engagement with preventive measures and treatments [31]. The paper have shown that most of the included studies reported significant differences in the TyG index between depressed and non-depressed patients. Specifically, some studies have found that the TyG index is significantly higher in depressed patients than in non-depressed patients. For example, Jin et al showed that individuals with depression had significantly higher mean TyG indices than those without depression (8.77 vs. 8.62, *p*<0.001) [32]. In addition, Lee et al reported similar results, with individuals with depressive symptoms having significantly higher TyG indices than those without depressive symptoms (4.70 vs. 4.64, *p* = 0.005). However, other studies have not found a significant association between depression and the TyG index. Overall, these studies suggest that the TyG index may be associated with depression, but more research is needed to further validate this association [33].

## Study strengths and limitations

Our study’s strengths include the use of a large sample size and robust statistical methods. However, limitations such as potential biases, sample size, study design, and data collection methods should be considered. The statistical insignificance of certain associations may be attributed to these factors, highlighting the need for further research to clarify these complex relationships.

## Clinical significance

Early diagnosis and intervention of prediabetes are crucial in preventing diabetic transformation and complications. Intensive lifestyle modifications such as diet, exercise, and weight loss are more beneficial than metformin use. Our findings indicate a positive association between depression and the prevalence of prediabetes, as well as increased all-cause mortality in prediabetic patients. This underscores the necessity for further research to explore the potential links between mental health and metabolic disorders. Our research report provides a new insight into the risk and treatment of prediabetes from the perspective of mental health conditions. Endocrinologists should focus on the mental health of patients with prediabetes, not just those with diabetes. Endocrinologists should not only pay attention to patients’ mental health status during diabetes treatment but also pay attention to preventing the development and worsening of prediabetes by paying attention to mental health status. Future research is warranted to investigate whether therapeutic interventions aimed at addressing mental health can reverse or impede the progression from prediabetes to diabetes, thereby preventing subsequent negative health outcomes.

## Conclusion

In conclusion, moderate to severe and severe depression was associated with the risk of prediabetes. Moderate to severe depression is positively associated with mortality in patients with prediabetes. This suggests that while there may be a trend towards relationships between depression and prediabetes, the evidence from this dataset is insufficient to draw a clear association. Further dose-response effect research is needed to establish the causal mechanisms underlying this association and to develop effective interventions that target both physical and mental health outcomes.
